# Investigation into antibacterial and wound healing properties of platelets lysate against *Acinetobacter baumannii* and *Klebsiella pneumoniae* burn wound infections

**DOI:** 10.1186/s12941-021-00442-x

**Published:** 2021-05-27

**Authors:** Aref Shariati, Alireza Moradabadi, Ehsanollah Ghaznavi-Rad, Maryam Dadmanesh, Majid Komijani, Farshad Nojoomi

**Affiliations:** 1 Microbiology Department, Faculty of Medicine, AJA University of Medical Sciences, Tehran, Iran; 2 Department of Medical Laboratory Sciences, Khomein University of Medical Sciences, Khomein, Iran; 3 Department of Medical Laboratory Sciences, School of Allied Medical Sciences, Arak University of Medical Sciences, Arak, Iran; 4 Infectious Diseases Research Center, Aja University of Medical Sciences, Tehran, Iran; 5 Department of Infectious Diseases, School of Medicine, Aja University of Medical Sciences, Tehran, IR Iran; 6 Department of Biology, Faculty of Science, Arak University, 38156-8-8349 Arak, Iran

**Keywords:** Burn wound infection, *A. baumannii*, *K. pneumoniae*, Platelets derived biomaterials

## Abstract

**Background and aim:**

Treatment of burn wound infections has become a global challenge due to the spread of multidrug-resistant bacteria; therefore, the development of new treatment options for the mentioned infections is essential. Platelets have drawn much attention for this purpose because they are a safe and cost-effective source of different antimicrobial peptides and growth factors. The present study evaluated antibacterial effects and wound healing properties of Platelet-derived Biomaterial (PdB) against *Acinetobacter baumannii* and *Klebsiella pneumoniae* burn wound infections.

**Methods:**

PdB was prepared through the freezing and thawing process and then, in vitro antibacterial effect was determined by disk diffusion and broth microdilution methods. Afterward, burn wound was inflicted on 56 rats, infected with both bacteria, and topical administration was performed to evaluate antibacterial effects and wound healing properties of PdB.

**Results:**

In vitro results showed that PdB inhibited the growth of *A. baumannii* in the highest dose (0.5), while we did not detect any inhibitory effects against *K. pneumoniae*. By contrast, PdB significantly inhibited the growth of bacteria in treated animal wounds compared to the control groups (*P* value < 0.05). Macroscopic assessments pointed to the significant enhancement of wound closure in the treated animals. In addition, histopathological examination demonstrated that treatment of rats with PdB led to a considerable increase in re-epithelialization and attenuated the formation of granulation tissue (*P* value < 0.05).

**Conclusion:**

The use of topical PdB is an attractive strategy for treating *A. baumannii* and *K. pneumoniae* burn wound infections because it inhibits bacterial growth and promotes wound healing properties.

## Introduction

Burn is identified as the fourth most common type of trauma worldwide that injures body tissues, especially skin [1]. Burn injuries lead to prolonged hospitalization and such patients are more likely to be infected with nosocomial pathogens. Notably, wound infections are caused by the microorganisms residing in patients’ gastrointestinal tracts or contaminated external sources such as hands of fomites, hospital environments, and healthcare workers’ hands [1, 2]. There is an increased risk of developing an invasive burn wound infection for burn patients such that bacteremia and multiple-organ dysfunction syndrome will develop. In this regard, burn wound infections, directly or indirectly, cause death by 33 to 80% [3, 4]. Many factors like breakdown of the skin barrier, immunodeficiency, inter-institutional transfer, and invasive procedures carried out at healthcare facilities make burn patients more susceptible to infections. Moist protein-rich eschar is the most common area that supports microbial growth because this area is avascular and prevents the delivery of immune cells and antibiotics [1, 5].

Emerging Multidrug-Resistant (MDR) bacteria have led to unexpected increase in the number of burn wound infections, hence sepsis and death worldwide [6]. In this regard, *Acinetobacter baumannii* and *Klebsiella pneumoniae* are recognized as some of the most frequent bacterial pathogens associated with MDR wound infections [7]. Clinical isolates of *A. baumannii* are also resistant to routine antibiotics; furthermore, using high toxicity drugs like tigecycline and colistin may induce many side effects in patients [8]. Therefore, the empirical antibiotic therapy employed to treat wound infections caused by this bacterial pathogen would not suffice, and incurable wound infections often lead to extensive surgical debridement and cause further or complete amputation of limbs and lengthen wound healing time duration [9]. Moreover, *K. pneumoniae* is also considered one of the primary nosocomial pathogens with the ability to colonize the human skin and mucosae and cause wound infection with severe local and systemic diseases [10]. Dissemination of antibiotic resistance, especially to carbapenem, fluoroquinolones, and colistin, is becoming increasingly severe, and only a few therapeutic options remain to treat MDR *K. pneumoniae* infections [11, 12]. Thus, MDR *A. baumannii* and *K. pneumoniae* usually complicate wound infection prognosis, a phenomenon that highlights urgent need for new therapeutic options against these bacterial pathogens.

In this respect, recent studies have used nanoparticles, bacteriophages, and natural products for preventing wound infection [13–15]. Our recent investigations have also shown that Platelet (PLT) derived biomaterials shorten wound healing time and prevent *Staphylococcus aureus* burn wound infections [16]. PLT is derived from patients’ blood and the production of cytokines and growth factors such as Transforming Growth Factor-beta (TGF_β_), PLT-derived growth factor, and Epidermal Growth Factor (EGF) can accelerate angiogenesis and wound healing process [17]. Furthermore, Tang et al. pointed to the direct antimicrobial role of PLTs as they are activated to release antimicrobial peptides such as platelet factor 4, connective tissue activating peptide 3, and RANTES, which would restrict microbial growth in the wound [18]. Therefore, the present study evaluated antibacterial effects and wound healing properties of PLT lysate against *A. baumannii and K. pneumoniae* burn wound infections.

## Material and methods

### Preparation of PLT material

In the present study, Platelet-derived Biomaterial (PdB) was prepared by freezing and thawing according to our recent research [15]. In brief, expired (one day; outdated) healthy human Platelet-Rich Plasma (PRP) was collected from the Blood Bank Center of Arak medical university. A number of platelets in the PRP were centrifuged in 150*g*, and the platelet-poor plasma was obtained from the supernatant. In this situation, PRP was concentrated with a platelet count of 2.5 × 10^12^/L. Notably, during PRP preparation, we used cell counter to measure the PLT count aseptically. The large number of platelets interrupts cell counter analysis and the dilution prevents aggregation. In this respect, a serial dilution (1/10, 1/100, 1/1000 dilution) of the PLT was used in the counting process to prevent aggregation in the cell counter. Every dilution counts and the count fold in dilution as well as the exact number of platelets were measured.

At the next steps, to prepare PdB, 90 mL of the collected PRP was activated by sterile calcium chloride (CaCl_2_) (25 mol/L). At this step, the coagulation cascade begins and thrombin and fibrin are produced. After that, the activated platelet is frozen at − 70ºC for 30 min and, subsequently, is thawed in 37ºC water bath for another 30 min. This process was repeated four times. The freezing and thawing method was employed to disrupt platelets’ membrane and release their internal content. In the end, after preparation, the suspension was centrifuged at 1400*g* for 15 min to remove the platelet lysate bodies. The supernatant without PLT bodies was collected by Pasteur pipette. This product (Freeze- PdB" (F-PdB)) was initially sterile-filtered next frozen in 1.5 to 2-mL aliquots at a temperature lower than − 20 °C.

### Bacterial strains

*A. baumannii* ATCC 27,853 and *K. pneumoniae* ATCC 700,603 were used in this study. All the culture media used in the present study were purchased from Merck (Germany) company. Bacterial strains were grown on the Brain Heart Infusion (BHI) broth medium overnight and then, were incubated for 18–24 h on fresh BHI medium at 37 °C until reaching the mid-logarithmic phase. The subculture was centrifuged at 1000*g* for 15 min and the supernatant was taken out. The bacterial pellet was then washed twice with Phosphate-Buffered Saline (PBS) and resuspended in cold PBS to achieve a concentration of approximately 1 × 10^8^ CFU/mL. The bacterial suspension was kept at − 20 °C until the next steps [19].

### Determination of antibacterial activity by Disk Diffusion (DD) and microdilution broth

After preparing a bacteria stock solution that contains 10^8^ CFU/mL bacteria, both strains were grown on the Muller Hinton Agar (MHA). Then, thin blank disks soaked with 30 μl of F-PdB were placed on the agar medium, and the plates were left to be incubated at 37 °C for 18–24 h. If the F-PdB inhibited the growth of bacteria or killed them, the zone of inhibition would be observed visually and reported according to the following criteria: no inhibition zone and inhibition zones of 0.5, 0.5–1, and greater than 1 mm [20].

The minimum inhibitory concentration of F-PdB was determined using micro-broth dilution assays on 96-well polypropylene tissue culture plates in line with the Clinical and Laboratory Standards Institute (CLSI) guidelines [21]. The proper concentration of each bacterium, 5 × 10^6^ CFU/mL, was prepared. Next, 90 μl of F-PdB was diluted on a fresh culture medium (ranging from 0.5 to 0.015), and 10 μl of bacterial suspension was added to each well. Positive control contained culture medium and bacteria without F-PdB. The test was performed in triplicate for each bacterial strain, and if the results of matched wells were different from each other, antibacterial activities were measured again. Upon overnight incubation, the last well in the series without any visible growth was determined as the Minimum Inhibitory Concentration (MIC) value and subculture for confirmations. Of note, plasma and CaCl_2_ were used as control at this stage [19, 22].

### Burn procedure

The experimental protocols of the present study were approved by the Ethics Committee on Animal Research of AJA University of Medical Science (Protocol number IR. AJAUMS. REC. 1399. 106). Fifty-six young male Wistar albino rats weighing 250–300*g* were randomly assigned to four groups of 14 rats, each. These groups include *A. baumannii* and *K. pneumoniae* controls that receive plasma after burn injuries (Control groups). Two other groups were treated by F-PdB after burn wound infection (Treated groups). All of the animals were obtained one week before the start of the experiment, had unrestricted access to water and food, and were housed in a suitable cage under controlled humidity, temperature, and 12-h light/dark conditions. On the day of wounding, electric clippers were used for shaving the dorsal hairs of rats and the target area was thoroughly disinfected. Then, rats were sedated by intramuscular injection of 10 mg/kg and 50 mg/kg of xylazine hydrochloride and ketamine, respectively. Calibrated bar at a temperature of 160 °C was applied to the dorsal shaved skin of the rats for 10 s to create a full-thickness burn wound. Afterward, animals in the control and treated groups were infected by10^8^ CFU/mL of *A. baumannii*, while the wound of the two other groups was inoculated with *K. pneumoniae*. One day after induction of burn wound infection, 1 mL of F-PdB was placed onto the wound of animals in treated groups, and due to the short lifetime of PLT, F-PdB was used every day for 14 days. After each treatment, to inhibit the cross-contamination, wounds were bandaged by Tegaderm [16, 19, 23].

### Measurement

On Days 4, 7, and 14 following the experiment, to evaluate the wound healing process, photographs were taken and the lesion area was measured (mm^2^) using the NIH Image J software according to the previous study [24]. Furthermore, on Days 4 and 7 post-infection, five rats in each group were killed and biopsy samples were collected aseptically; then, they were weighed and homogenized in 10 mL PBS and inoculated into MHA. Besides, colony count was reported as log_10_ CFU/gram tissue. Notably, punch biopsy specimens from the wound area on the mentioned days were fixed in 10% formalin and paraffin-embedded materials and, then, were processed for Hematoxylin and Eosin (H&E) staining. In this manner, control and treated animal groups were compared to each other by a pathologist (who was blinded to animal and treatment groups) based on re-epithelialization and the thickness of granulation tissue. Finally, the specific parameter-covered areas (%) per high power field were presented as parameter percentages [16, 19].

### Statistical analysis

Findings were analyzed using the Mann–Whitney test when data were non-normally distributed, and statistical analyses were performed using GraphPad Prism (ver. 8.3.0) and SPSS (ver. 20.0) (SPSS Inc. Chicago, IL, USA). The difference between the treated and control groups was statistically significant when the *P* value was lower than 0.05.

## Results

### In vitro antibacterial activity of F-PdB

F-PdB had an inhibitory effect against *A. baumannii* in DD and broth microdilution methods. In this regard, the inhibition zone (1 mm) of this bacterium was observed in the presence of the disks soaked with the F-PdB. Furthermore, the results of using broth microdilution methods showed that F-PdB inhibited the growth of *A. baumannii* at the highest concentration (0.5). On the other hand, our findings did not show any in vitro antibacterial effects for F-PdB against *K. pneumoniae*. Notably, plasma and CaCl_2_ used as controls did not show any inhibitory effect.

### In vivo antibacterial activity of F-PdB

**Fig. 1:**
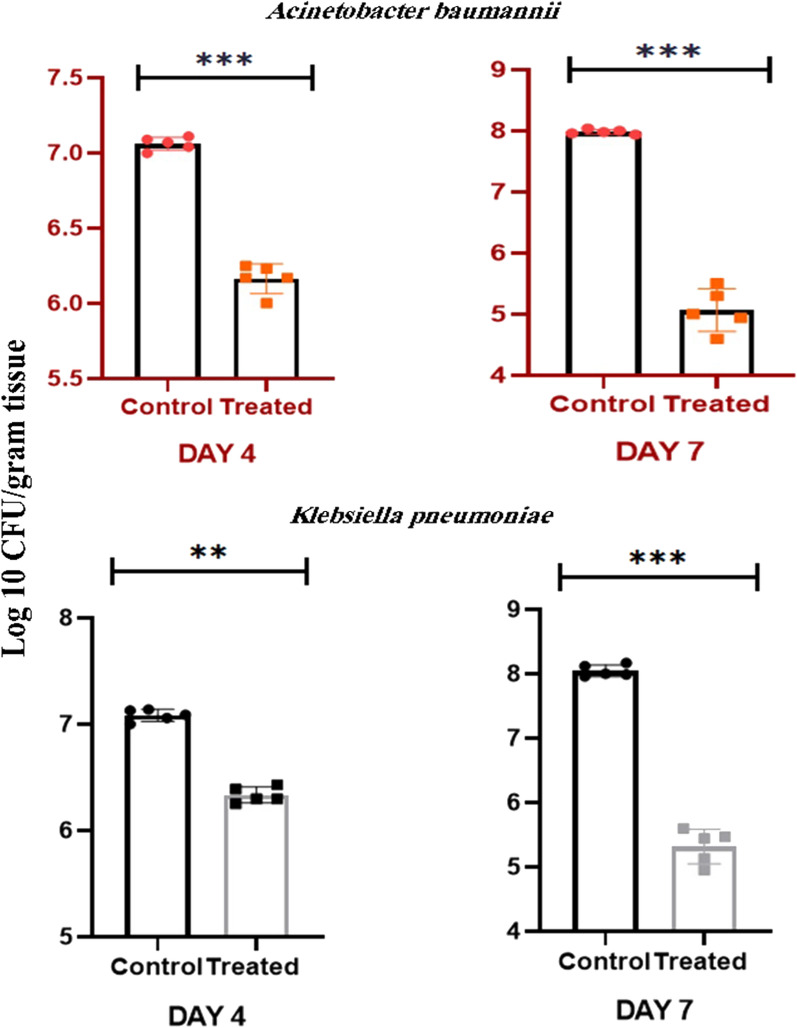
Wound burden of *A. baumannii* and *K. pneumoniae* on Days 4 and 7 in rats infected with 1 × 10^8^ cells of each bacterium determined by the amount of CFU growth (n = 5 wounds per group). F-PdB treatment significantly decreased the number of bacteria in the treated group compared to the control group. **P value < 0.01, ***P value < 0.001

Biopsies were collected from the wound on Days 4 and 7 and, then, homogenized in PBS. Quantified bacteria were reported on log_10_ CFU/gram tissue. Rats in the control group were subject to the largest number of bacteria, while in the treated groups that included animals treated with F-PdB, the number of *A. baumannii* and *K. pneumoniae* was significantly reduced (*P* value < 0.05) (Fig. 1). Notably, blood parameters such as white blood cells, Hematocrit, and hemoglobin were monitored continuously during the experiment, and no signs of blood infections were observed in animals of each group.

### Wound healing properties of F-PdB

**Fig. 2:**
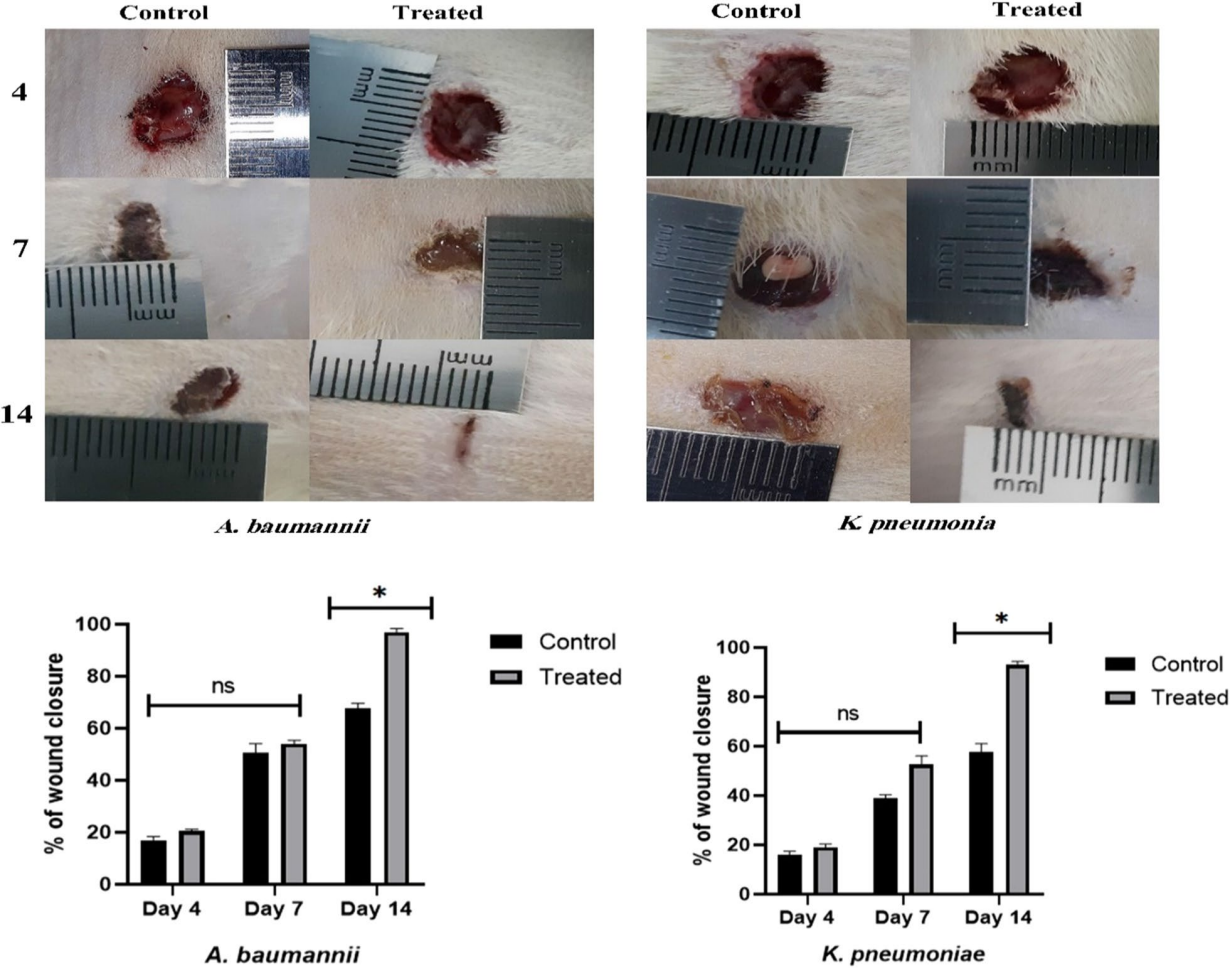
Wound closure degree in various animal groups. Macroscopic evaluation of *A. baumannii* and *K. pneumoniae* burn wound healing was done for the treated group with F-PdB and the control group. Insignificant changes in the wound closure degree were observed in the F-PdB and control groups on the 4th and 7th days (P > 0.05), while on the 14th day after wounding, the mean size of the burn wound area was significantly smaller in the animal treated with PdB than that in the control groups (P < 0.05)

**Fig. 3:**
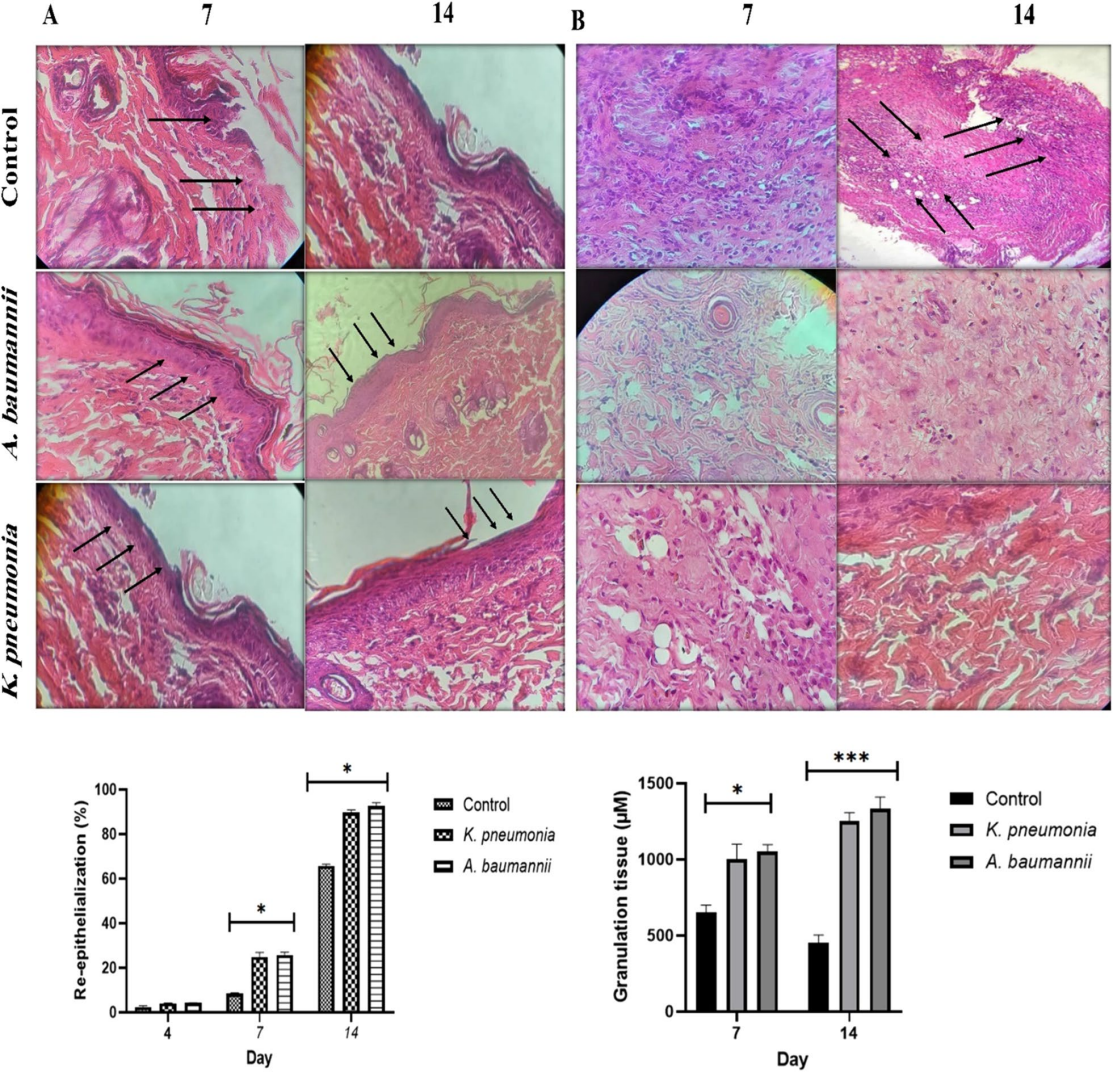
Histopathologic examination of burn wound infection. Cross-sectional biopsy samples were collected on Days 4, 7, and 14 from *A. baumannii* and *K. pneumoniae*-infected rat burn wounds; then, H&E staining was conducted. Treatment with PdB accelerated the wound healing process. **A** There is no significant difference in the re-epithelialization rate of the PdB-treated and control groups on Day 4. This treatment increased re-epithelization remarkably in the treated animal compared to the control groups on Days 7 and 14. **B** wide granulation tissue was observed in PdB-treated animals in comparison to that in the control groups. Data are presented as mean ± SEM. *P value < 0.05, ***P value < 0.001

Macroscopic photographs were taken every day per treatment and the treated animals experienced better wound healing than those in the control group. In addition, a significant amount of pus was observed in the control rats infected with *K. pneumoniae* (Fig. 2). Totally, statistical analysis showed a significant increase in the percentage of wound closure with F-PdB in the treated groups (*P* value < 0.05). Besides, histological examination confirmed the existence of a third-degree burn (full-thickness burns) in all animals in different groups after the experiment. Moreover, assessments of biopsies collected from the wound on Days 4, 7, and 14 showed that re-epithelization was significantly higher in the treated group than that in the control group on the 7th and 14th days, while no significant differences were identified on the 4th day. Of note, in the first seven days, initial acceleration of epithelial formation was observed in the treated groups, compared to the control animals. Furthermore, complete re-epithelialization was observed in treated animals on Day 14. In additions, histopathologic evaluations demonstrated that granulation tissue was less developed in control animals than in the PdB-treated group (Fig. 3).

## Discussion

Burn wound infection is considered a major therapeutic challenge against managing burn wounds, and it causes morbidity and mortality around the world. The use of antibiotics to control wound infection remains limited due to increase in the prevalence of MDR bacteria. Furthermore, systemic antibiotics therapy may not be able to prevent wound infection due to different factors like necrosis, granulation tissue, limited peripheral blood supply, and fibrosis that inhibit the penetration of antibiotics into the burned tissues. Therefore, topical use of antibacterial agents can be effective in controlling burn wound infection [14, 19, 25]. In this context, in the present study, F-PdB was used for the inhibition of *A. baumannii* and *K. pneumoniae* burn wound infection. In vitro results showed that F-PdB inhibited the growth of *A. baumannii*. In a previous study performed by Tang et al., normal human platelets were stimulated with human thrombin, and purified peptides showed antibacterial effect against *Escherichia coli* and *S. aureus* [18]. Therefore, direct inhibitory and antibacterial effects of F-PdB may be related to different antimicrobial proteins and peptides. However, we did not detect inhibitory effects against *K. pneumoniae.* A polysaccharide matrix that coats the bacteria, capsule, is one of the most important virulence factors of *K. pneumoniae*. Capsule hinders phagocytosis and opsonophagocytosis, prevents the antibacterial activities of antimicrobial peptides by binding these molecules distant from the outer membrane, and inhibits complement-mediated lysis and opsonization by blocking complement components [26]. Thus, this virulence factor may lead to F-PdB resistance of *K. pneumoniae*, although a previous study reported that PRP, solvent/detergent-treated PLT lysate, and PLT-rich plasma inactivated *K. pneumoniae* ATCC 13,883 [20]. These differences could result from intrinsic characteristics of the bacterial strains used and the use of different methods of PLT preparation and platelet concentrates [27]. Nevertheless, further studies are needed to determine the exact antibacterial mechanism of PLT-derived biomaterials.

In the current study, to simulate a real clinical situation, we used rat burn wound infection model. Our results indicated that the use of F-PdB was beneficial for the topical treatment of infected wounds, and in addition to reducing the number of bacteria in the wound, it also induced faster wound closure and accelerated the healing process of burn wounds. Contrary to in vitro results, *K. pneumoniae*, like *A. baumannii*, was significantly inhibited in wounds; thus, it seems that F-PdB can have an excellent antibacterial effect by stimulating the host immune system. In this context, a recent study have also reported that PRP could accelerate the healing process in the deep second-degree burns associated with diabetes mellitus, but not in third-degree burns in rats [23].

Moreover, Ozcelik et al. reported the higher Hydroxyproline levels and the lower inflammatory cell infiltration in the animals treated with PRP than the control group [28]. Our recent studies have also shown that PLT-derived biomaterial inhibits methicillin-resistant *S. aureus* burn wound infection and lymphocutaneous sporotrichosis [16, 29].

Therefore, PLTs have a potential role in contributing to wound healing processes. In this manner, recent studies have reported that different growth factors released by PLT such as TGF_β_ and fibroblast, epidermal, keratinocyte, and vascular endothelial growth factors have specific roles in wound healing and tissue regeneration. These factors elevate angiogenesis, proliferation of mesenchymal and epithelial cells, and vascular permeability. Besides, collagen synthesis, epithelialization, and endothelial cell migration/proliferation are induced by these factors [30, 31]. Of note, PLT recruits inflammatory cells to the zone of injury by production mediators such as CXC Chemokine Ligand-4 (CXCL-4) and Platelet Factor 4 (PF4). Furthermore, integrin receptors and P-selectin on the surface of activated cells mediate the interaction of PLT with neutrophil and macrophage, upregulate inflammatory cell recruitment, and facilitate progression into the inflammatory phase of wound healing [32, 33].

Therefore, better inhibition and killing of bacterial pathogens in in vivo than in the in vitro conditions showed that the antibacterial effects of PLT alone were negligible, which could be sustained through a co-operation of plasma components and platelet-derived factors [27, 34]. Similarly, Burnouf et al. reported that inactivating the complement using heat would cause platelets to lose their antimicrobial activities [20]. Another study also confirmed these results and found that plasma components had an essential role in the antimicrobial activity of P-PRP [34]. As mentioned, various studies have reported that PRP can inhibit bacterial pathogens and accelerate wound healing. However, in the present study, we used PLT lysate which lacked PLT bodies. Indeed, PRP was shown to induce an intense inflammatory response, as manifested in the production of granulation tissue. The increased inflammatory phase can stimulate the formation of hypertrophic scarring, which needs to be avoided due to superficial partial-thickness defects [35, 36]. So, we used PLT lysate for the treatment of burn wound infection to hinder immunological reactions that may be caused by platelet bodies.

## Conclusion

The results of current research showed that F-PdB was beneficial for the topical inhibition of *A. baumannii* and *K. pneumoniae* burn wound infection; furthermore, it caused faster wound closure and accelerated wound healing. Thus, PdB can be a good treatment option for the inhibition of burn wound infections due to its widespread and topical use (a small amount of gel or liquid), and its concentration increased at the target site compared to systemic antibiotic therapy. Furthermore, topical treatment of wound infections reduced the risk of side effects like neuropathy, gastrointestinal disturbances, and nephropathy. However, more researches are needed to evaluate the antibacterial effects of PdB for inhibition of MDR bacteria and multi-species wound infection. Moreover, the anti-biofilm properties of PdB should be assessed because biofilm-associated infections are remarkably resistant to clearance by the host immune system and antimicrobial therapy.

## Data Availability

Data sharing not applicable to this article as no datasets were generated during the current study.
